# Radiopharmaceutical Stem Cell Tracking for Neurological Diseases

**DOI:** 10.1155/2014/417091

**Published:** 2014-05-25

**Authors:** Paulo Henrique Rosado-de-Castro, Pedro Moreno Pimentel-Coelho, Bianca Gutfilen, Sergio Augusto Lopes de Souza, Gabriel Rodriguez de Freitas, Rosalia Mendez-Otero, Lea Mirian Barbosa da Fonseca

**Affiliations:** ^1^Department of Radiology, School of Medicine, Universidade Federal do Rio de Janeiro, Rio de Janeiro, RJ, Brazil; ^2^Instituto de Biofísica Carlos Chagas Filho, Universidade Federal do Rio de Janeiro, Avenida Carlos Chagas Filho 373, CCS, Bloco G, Cidade Universitária, Ilha do Fundão, 21941-590 Rio de Janeiro, RJ, Brazil; ^3^D'Or Institute for Research and Education, Rio de Janeiro, RJ, Brazil

## Abstract

Although neurological ailments continue to be some of the main causes of disease burden in the world, current therapies such as pharmacological agents have limited potential in the restoration of neural functions. Cell therapies, firstly applied to treat different hematological diseases, are now being investigated in preclinical and clinical studies for neurological illnesses. However, the potential applications and mechanisms for such treatments are still poorly comprehended and are the focus of permanent research. In this setting, noninvasive *in vivo* imaging allows better understanding of several aspects of stem cell therapies. Amongst the various methods available, radioisotope cell labeling has become one of the most promising since it permits tracking of cells after injection by different routes to investigate their biodistribution. A significant increase in the number of studies utilizing this method has occurred in the last years. Here, we review the different radiopharmaceuticals, imaging techniques, and findings of the preclinical and clinical reports published up to now. Moreover, we discuss the limitations and future applications of radioisotope cell labeling in the field of cell transplantation for neurological diseases.

## 1. Introduction

In spite of the significant progress achieved in the medical field in the past decades, neurological illnesses remain as one of the leading causes of disease burden in the world [1, 2]. In the next years, with the progressive ageing of the population, the prevalence of these diseases and the expenses associated with them are expected to increase even more [2]. Contemporary treatments such as pharmacological agents are restricted in their potential to improve neurological function and are unable to promote restoration of lost neurons and other brain cells damaged in such diseases. Stem cell transplantation, initially developed more than 40 years ago to treat hematological malignant disorder, has more recently demonstrated promising results in different ailments, including autoimmune, cardiovascular, and neurological diseases [3, 4].

The use of noninvasive* in vivo* imaging to track the transplanted cells allows a better understanding of several aspects of cell therapies, including their biodistribution. In this scenario, radioisotope cell labeling, an already well-established nuclear medicine technique, has emerged as one of the most powerful tools. In the following sections, we will review the preclinical and clinical studies that used radiopharmaceutical stem cell tracking for neurological diseases and discuss important aspects in the area.

## 2. Radiopharmaceutical Cell Labeling

Radiopharmaceutical cell labeling has been used for decades to systemically monitor cells in nuclear medicine studies such as labeled leukocyte scintigraphy for detection of infectious and inflammatory diseases [5–7]. Technetium-99m ( ^99m^Tc) is currently the most used radionuclide in the world and is imaged with conventional nuclear medicine techniques, that is, 2-dimensional planar scans or 3-dimensional single photon emission computed tomography (SPECT). Additionally, SPECT images may be fused with conventional computed tomography (CT), resulting in SPECT/CT images that allow attenuation correction and better localization of nuclear medicine findings, significantly improving both sensitivity and specificity [8].  ^99m^Tc has wider availability and lower cost than other radionuclides, and its 6-h physical half-life allows cell tracking for up to 24 h with good resolution and low radiation dose to the patient and to the labeled cells [5–7].

Another conventional nuclear medicine radiopharmaceutical indium-111-oxine (^111^In-oxine) allows cell tracking for up to 96 h but results in lower resolution images and leads to higher radiation dose to the patient and to the labeled cells. In addition, different studies have indicated that Auger electrons of ^111^In-oxine labeling affect cellular integrity and lead to cytotoxicity of stem cells [9–12]. ^18^F-FDG, which has a 110-minute half-life, is the most commonly used radiopharmaceutical for positron emission tomography (PET) and allows cell labeling and tracking for a few hours. Unlike SPECT scans, which are less commonly acquired with hybrid CT equipment, PET is routinely made in scanners that allow PET/CT acquisition. Moreover, PET has a two- to threefold higher spatial resolution than SPECT (3–6 mm versus 10–15 mm) and allows quantification of standardized uptake values, which may be used to compare response to different therapies [13–15].

Stem cell tracking with SPECT and PET may be separated in two strategies: direct and indirect. Direct labeling is made by incubating stem cells with a radiotracer* in vitro* and subsequently transplanting them and can be done with radiopharmaceuticals such as  ^99m^Tc-hexamethylpropyleneamine oxime ( ^99m^Tc-HMPAO) or ^111^In-oxine for SPECT and ^18^F-FDG for PET. Indirect cell labeling may be carried out via reporter gene/probe systems, which has been subject to excellent reviews [16–18]. In brief, reporter gene/probe systems have traditionally been divided in three groups, according to the way that the protein product of the reporter gene interacts with the reporter probe and causes its accumulation on the surface or inside the cells [16–18]: (1) reporter genes that encode enzymes that phosphorylate specific reporter probes leading to their entrapment; (2) reporter genes that encode protein receptors which in their turn bind to specific reporter probes; and (3) reporter genes that encode cell membrane transporters that accelerate the accumulation of reporter probes in the cells. One example of a reporter gene/probe system is the herpes simplex virus type I thymidine kinase (HSV1-TK) reporter gene that catalyzes reactions leading to the entrapment of a probe such as iodine-131-2′-fluoro-2′-deoxy-5′-iodo-1*β*-D-arabinofuranosyluracil (^131^I-FIAU) for SPECT.

## 3. Search Strategy

For this review, references were identified in MEDLINE databases using the terms “nuclear medicine,” “cell therapy,” “labeled,” “neurologic,” and “brain.” Only articles published in English were reviewed and no date restriction was made.

## 4. Published Preclinical Trials

We identified 17 published articles in English that used radiopharmaceuticals to track the biodistribution of transplanted cells in animal models of neurological diseases. Eight of these were performed in a model of transient middle cerebral artery occlusion (MCAO), 3 in traumatic brain injury, 3 in global brain ischemia, two in spinal cord lesion, and one in thermocoagulation focal brain ischemia. Seven studies used ^111^In-oxine, another 7 utilized  ^99m^Tc or  ^99m^Tc-HMPAO, two employed ^131^I-FIAU, and ^18^F-FDG was used in one (Table 1). Of note, eight (47%) of these studies were published in 2013.

### 4.1. Spinal Cord Injury

de Haro et al. [19] investigated radiopharmaceutical cell tracking 3 months after compressive spinal cord injury at T6-T8 level in rats. They injected 6 × 10^6^ rat bone marrow derived mesenchymal stem cells (BM-MSCs) labeled with ^111^In-oxine by the tail vein or by intralesional injection and performed whole-body planar imaging from 3 to 10 days. They found that intravenous injection of ^111^In-oxine labeled BM-MSCs led to biodistribution mainly to the spleen, liver, and kidneys, while the vertebral column showed faint migration and the spinal cord did not show any activity. Also, there was high activity in the tail, where the cells were injected. In contrast, when the ^111^In-oxine labeled cells were injected into the traumatic centromedullar cavity, there was a persistent homing into the lesion site, without any distribution to the rest of the organism in the 10-day imaging period.

Lo et al. [22] carried out the transplantation of mouse embryo-derived fibroblasts from lineage NIH3T3 in a rat model of compressive spinal cord injury at T10 level. These cells contained the HSV1-TK reporter gene. A total of 1 × 10^6^ cells labeled with ^131^I-FIAU were injected at the L1 level immediately after the spinal cord lesion and whole-body planar imaging was performed at 2, 24, and 48 h. After cell transplantation, uptake was observed in the injection site at all time points.

### 4.2. Traumatic Brain Injury

Yoon et al. [24] explored the migration of rat BM-MSCs labeled with ^111^In-oxine injected in rats subjected to traumatic brain injury and in sham-operated animals. A total of 1 × 10^6^ cells were injected in the tail vein 24 h after the lesion and whole-body planar imaging was performed 24 h later. The viability and proliferation of labeled BM-MSCs were assessed for 14 days. In the whole-body images, the majority of ^111^In-BM-MSC signal was detected in the liver and spleen. Uptake in the brain was higher in animals that suffered traumatic brain injury (1.4%) in comparison to sham-operated (0.5%) or normal rats (0.3%).

In another study by the same group, Park et al. [25] tracked  ^99m^Tc-HMPAO-labeled rat BM-MSCs transplanted in rats with traumatic brain injury and in sham-operated animals. Approximately 1 × 10^6^ cells were infused in the tail vein 24 h after the lesion. Whole-body images were made 4 h later and subsequently the animals were euthanized and tissue samples were collected for gamma well counting. The authors also performed cytometric evaluation to assess apoptotic or necrotic changes up to the 7th day after labeling. Gamma well counting indicated that homing to the liver, kidneys, lungs, and spleen was not different between traumatic brain injury (36.7% ± 6.9%, 16.1% ± 1.7%, 8.2% ± 0.7%, and 2.6 ± 0.7%, resp.) and sham-operated rats (41.2% ± 6.7%, 17.4% ± 2.1%, 8.1% ± 0.9%, and 2.9 ± 1.0%, resp.; all *P* > 0.05). In spite of that, brain signal in traumatic hemispheres was significantly higher than in contralateral hemispheres, and this did not occur in sham-operated animals. Notwithstanding, on whole-body images there was no significant uptake in the brains of traumatic animals or controls.

Guan et al. [35] performed the only study using ^18^F-FDG for stem cell labeling in a neurological disease. Rats submitted to traumatic brain injury received human BM-MSCs only or collagen scaffolds impregnated with human BM-MSCs. A total of 3 × 10^6^ ^18^F-FDG labeled cells were used in each group and whole-body PET imaging was carried at 3, 6, and 12 h after cell transplantation. At the end of the imaging experiments, rats were euthanized and tissue samples from the two cerebral hemispheres, cerebellum, heart, lungs, liver, spleen, and kidneys were obtained for gamma-well counting. In whole-body scans at 6 and 12 h, animals that received the collagen scaffolds with BM-MSCs showed higher uptake in the lesion cavity and lower uptake in the contralateral hemisphere and other organs in comparison with animals that received BM-MSCs only. Furthermore, this finding was confirmed in gamma-well counting at 12 h after transplantation.

### 4.3. Transient Middle Cerebral Artery Occlusion

Mäkinen et al. [20] evaluated the homing of human umbilical cord blood mononuclear cells labeled with ^111^In-oxine 24 h after 120 minutes of transient MCAO in rats, one of the most commonly used models of stroke. An infusion of 1–7 × 10^6^ cells was carried out in the femoral vein and SPECT/CT imaging was performed after 30 minutes and 24 h. The authors found that accumulation was greater in the lungs at 30 minutes and at 24 h in the liver. Homing of ^111^In-labeled cells was also seen in the spleen and kidneys, but not in the brain. Immunohistochemistry for human nuclei showed the presence of few cells in the brain, mainly in the ipsilateral hemisphere close to blood vessels.

Lappalainen et al. [21] examined the biodistribution of human embryonic stem cell-derived neural progenitor cells or rat hippocampal progenitor cells after 120 minutes of transient MCAO in rats. An injection of 1 × 10^6^ cells was made in the carotid artery or femoral vein 24 h after the lesion and SPECT/CT imaging was done at 0 and 24 h. After the last images, tissue samples from the liver, spleen, lungs, kidneys, and brain were analyzed in a gamma well counter. The authors found that intravenous infusion led to accumulation of transplanted cells mainly in the liver, spleen, and kidneys, respectively, with no visible uptake in the brain. On the other hand, after carotid artery injection a low uptake was seen in the brain, with the remaining uptake in the other internal organs. Furthermore, the authors estimated the detection sensitivity of SPECT/CT to be of about 1000 ^111^In-oxine labeled cells and that labeling did not influence cell viability.

Detante et al. [23] studied the migration of human BM-MSCs after 90 minutes of transient MCAO in rats and in a control group without cerebral ischemia. A mean of 3.4 ± 1.2 × 10^6^ cells were injected into the saphenous vein 7 days after the ischemia and planar imaging was performed after 2 and 20 h. Animals were euthanized after both time points and samples from different tissues were analyzed by gamma well counting. Whole-body imaging indicated a trend toward higher uptake in the brain in the ischemic group in comparison to control animals at both time points. Significant lung trapping occurred in all rats at 2 h but decreased at 20 h. Gamma well counting indicated higher activity in the ischemic hemisphere in comparison to the control group at 20 h. The authors estimated that 1 out of 10.000 cells migrated to the ischemic hemisphere. Strikingly, the uptake in the spleen increased between 2 and 20 h, while in the other organs it decreased. This finding could indicate that labeled human MSCs are sequestered in the spleen.

Arbab et al. [27] labeled human umbilical tissue-derived cells with ^111^In-oxine and injected them in the tail vein 48 h after transient MCAO in rats for 120 minutes. A total of 3 × 10^8^ cells were used and SPECT imaging was performed at 0, 1, and 3 days. The authors found that the majority of the cells were retained in the lungs on day 0 (43.36 ± 23.07%), while on days 1 and 3 this number declined to 8.81 ± 7.75 and 4.01 ± 4.52%, respectively. On the contrary, uptake in the lungs of rats that were given ^111^In-oxine alone persisted fairly unaltered from day 0 (18.38 ± 5.45%) to day 1 (12.59 ± 5.94%) and fell to 8.34 ± 4.25% on day 3. They also reported that the ratio between the ischemic and nonischemic hemispheres was significantly higher (*P* < 0.05) on days 0, 1, and 3 in the animals that were given ^111^In-oxine labeled cells (1.24 ± 0.29, 1.63 ± 0.39, 1.75 ± 0.59, resp.) compared to the animals that were given only ^111^In-oxine (0.95 ± 0.18, 1.13 ± 0.22, 1.16 ± 0.29, resp.).

Mitkari et al. [28] assessed the distribution of ^111^In-oxine labeled human BM-MSCs after 90 minutes of transient MCAO in rats and in sham operated animals. In addition to these two groups, they also analyzed the distribution of BM-MSCs treated with a proteolytic enzyme named Pronase (Roche, Mannheim, Germany), instead of trypsin, to transiently modify cell surface proteins. The rationale for the use of the proteolytic enzyme was that in previous unpublished data the authors saw a decrease in lung entrapment of healthy mice after intravenous injection of unspecified cells. Approximately 0.5 × 10^6^ (Pronase detached) to 1.1 × 10^6^ cells (trypsin detached) were injected in the external carotid artery 24 h after the injury and SPECT/CT images were acquired at 30 minutes and 24 hours. After the last scan animals were euthanized and samples from the brain, liver, lungs, spleen, and kidneys were collected for gamma well counting. At 30 minutes after cell transplantation, there was high uptake in the brain in both MCAO and sham-operated animals. Nevertheless, the cells seemed to relocate to the liver and other organs at 24 h. Gamma well counting indicated higher uptake in the ischemic hemisphere in MCAO rats. There was no significant difference in the homing between Pronase and trypsin treated cells.

Goldmacher et al. [30] explored the homing of ^111^In-oxine labeled rat BM-MSCs in rats after 120 minutes of transient MCAO and in sham operated animals. A total of 5 × 10^6^ cells were injected in the tail vein 24 h after the ischemia and whole-body planar images and brain pinhole images were acquired after 4, 20, 44, and 70 h. The authors described that the majority of intravenously infused cells remained in the lungs, while the uptake in the liver and spleen increased over time. Moreover, they described that in 44 and 70 h images higher activity was seen on the lesioned side of the head. However, no quantification was made in the images or by gamma well counting.

Manley et al. [32] tracked rat bone marrow dendritic cells (BM-DCs) labeled with  ^99m^Tc-HMPAO transplanted after a transient MCAO for 60 minutes in rats. About 2 × 10^6^ cells were injected in the carotid artery 3 h after the injury and SPECT images were performed at 5 to 20 minutes and 5 to 6 hours after transplantation. At 5 minutes after injection, 31% of the labeled BM-DCs homed to the lungs, 11% to the lesioned side of the head, and 26% to the liver, spleen, and gut. After 6 hours, 2–5% of the labeled BM-DCs remained in the lungs, 2-3% in the lesioned side of the head, and the remaining activity was found in the liver, spleen, and gut.

Wu et al. [34] analyzed the migration of rat BM-MSCs after 60 minutes of transient MCAO in rats. Cells were first transfected with the HSV1-TK reporter gene. Then, a total of 2 × 10^6^ cells were injected 24 h after the lesion by one of the 4 following routes: intracerebral, intraventricular, carotid artery, or tail vein. ^131^I-FIAU was injected subsequently and the animals underwent whole-body planar imaging at 2, 8, and 24 h. At 24 h, the animals were euthanized and samples from different tissues were obtained for gamma well counting. The authors reported that in whole-body images it was possible to visualize the liver and bladder and that the signal in the brain was small but augmented gradually over time. In gamma well counting, the uptake in the lesioned hemisphere was greater than in the contralateral hemisphere in MCAO animals but not in controls. Cell homing to the lesioned hemisphere was greater in animals that received intracerebral injection in comparison to the other routes.

### 4.4. Focal Cerebral Ischemia

Vasconcelos-dos-Santos et al. [26] conducted experiments after labeling bone marrow mononuclear cells (BM-MNCs) with  ^99m^Tc for cell therapy in rats subjected to focal cerebral ischemia by permanent thermocoagulation of pial blood vessels or in sham-operated animals. A total of 3 × 10^7^ cells were injected in the common carotid artery or in the jugular vein 24 h after the lesion or sham operation and whole-body imaging was carried out 2 and 24 h later. Animals were euthanized at 2 or 24 h and tissue samples were collected for gamma well counting. Whole-body images at 2 h showed high uptake in the head after intra-arterial infusion in ischemic and sham-operated groups, but not following intravenous administration of the cells. High uptake in the lungs, liver, and kidneys was also seen at 2 h. Gamma well counting at 2 h confirmed similar biodistribution between all groups, with high activity in the liver and lungs. Interestingly, the uptake seen in the head of the intra-arterial group was not due to signal in the brain. A possible reason for this finding could be that cells were dispersed along the tissues irrigated by the external carotid artery, such as the nasopharynx area. At 24 h, whole-body images showed that the remaining signal was seen mainly in the liver and kidneys, with a sharp decrease in lung uptake. Moreover, the uptake present in the head of intra-arterial groups at 2 h was no longer seen. These findings were also confirmed in gamma well counting. Although the uptake in the brain continued being low, the ischemic hemisphere had higher activity than the contralateral hemisphere, both for intravenous and intra-arterial groups.

### 4.5. Global Brain Ischemia

In a paper from our group, Gubert et al. [29] carried out the transplantation of rat BM-MNCs labeled with  ^99m^Tc in a rat model of global cerebral ischemia by bilateral common carotid artery ligation. About 2 × 10^7^ cells were infused in the tail vein 24 h after the injury and whole-body planar imaging was performed 1 h later. Liver and spleen were the organs with highest signal. Uptake in the head was similar to that of the other organs and soft tissues.

Makela et al. [31] performed cell therapy using pig BM-MNCs labeled with  ^99m^Tc-HMPAO in a model of global brain ischemia. In this model, pigs underwent transient brachiocephalic trunk and subclavian artery occlusion for 7 minutes. Approximately 6 to 20 × 10^6^ cells were injected in the brachiocephalic trunk 24 h after the lesion and whole-body planar imaging and head SPECT/CT imaging was made 2, 4, 6, 12, and 24 h later. Animals were euthanized at the different time points and tissue samples were obtained for gamma well counting. The mean uptake for all time points was quantified in whole-body images and showed that the lungs had the highest signal (32.7%), followed by the liver (14.2%), spleen (7.3%), and kidneys (2.5%). There was no significant uptake in the brain in whole-body images or gamma well counting.

Ramos et al. [33], from our group, studied the migration of rat BM-MNCs after cell transplantation in rats with transient global ischemia and in sham-operated controls. The lesion was produced by permanent vertebral ligation and transient carotid artery occlusion for 17 minutes. Then, 24 or 72 h after the ischemic insult, 3 × 10^7^ cells were infused in the common carotid artery. Whole-body planar imaging and SPECT/CT were performed 2 h later. Subsequently, animals from the group that received cells at 72 h after the lesion were euthanized and their brains were isolated for gamma well counting. On planar and SPECT/CT images, activity was observed mainly in the site of injection, liver and spleen, with no significant activity in the brain. Gamma well counting indicated higher activity in the brains of ischemic animals, indicating that, although in very low amount, global ischemic lesion increased the homing of  ^99m^Tc-BM-MNCs to the brain.

## 5. Published Clinical Trials

There have been 5 published articles in English concerning 2 different trials that used radiopharmaceuticals to track stem cell transplantation for neurological diseases, with a total of 13 treated patients (Table 2).

### 5.1. Acute Ischemic Stroke

Correa et al. [36] published a case report of a patient that received  ^99m^Tc-HMPAO labeled BM-MNCs after an ischemic stroke in the posterior branch of the left middle cerebral artery. A total of 3 × 10^7^ cells, 1% of them labeled, were injected in the left middle cerebral artery 9 days after the infarct. A whole-body planar scan and SPECT of the head were performed 8 h later. The SPECT scan showed homing of labeled cells to the anterior branch of the left middle cerebral artery, what probably occurred due to the persistent occlusion of the posterior branch. Whole-body images showed that uptake in the remaining organs occurred mainly to the liver and spleen. Nevertheless, no quantification of cell uptake in the different organs was made.

### 5.2. Subacute Ischemic Stroke

Our research group published a series of articles on a trial that used  ^99m^Tc labeled cells for subacute middle cerebral artery stroke [37–40]. A total of 1 to 5 × 10^8^ cells were infused in the middle cerebral artery or in the cephalic vein. Whole-body planar imaging was performed at 2 and 24 h and a SPECT of the head was carried out at 2 h after transplantation. Additionally, CT or MRI were performed before and up to 180 days after the therapy and the fusion of SPECT and CT or SPECT and MRI was performed. Seven patients were included in the intra-arterial group and 5 in the intravenous group, between 19 and 89 days after the stroke. The quantification of whole-body scans showed that the intra-arterial injection led to higher signal in the liver and spleen and lower signal in the lungs at 2 h when compared with the intravenous route. The analysis of SPECT images showed that the relative uptake in the ipsilateral hemisphere in comparison to the uptake in the brain was also higher in the intra-arterial group. At 24 h, the intravenous group had an increase in the percentage of homing to the liver and spleen and a decrease to the lungs when compared to 2 h scans. Nonetheless, the signal in the brain compared to the whole body continued being low and similar between both groups at 2 and 24 h.

## 6. Discussion

In addition to radiopharmaceutical cell labeling, there are other methods being investigated to track stem cells. For example, bioluminescence imaging has been successfully used to label cells in different models of stem cell therapies for neurological diseases [41–45]. However, it has narrow depth penetration (~1 cm) and cannot be used clinically [46]. Superparamagnetic iron oxide nanoparticles (SPIOs), initially developed for detection of hepatic lesions after intravenous injection, have also been used for cell labeling. In preclinical models, cell labeling with SPIOs allows tracking for days or weeks after injection with excellent resolution and anatomical correlation with MRI [47–49]. A few clinical trials have successfully labeled different stem cells with SPIOs for neurological diseases [50–53]. Nevertheless, SPIO labeling suffers from common limitations to other exogenous contrasts, such as dilution of the contrast media with cell division and the possibility that apoptotic stem cells may be phagocytized by macrophages, leading to signal that could be incorrectly associated with the cells. Furthermore, there are conflicting reports on the impact of nanoparticles in cellular events [54–57], and health authorities have not yet approved the use of SPIOs for cell labeling.

For these reasons, radiopharmaceutical cell tracking remains an important tool for evaluation of stem cell migration and homing. In addition to a more precise localization of the site of homing, the fusion of nuclear medicine images with CT or MRI allows evaluation of different aspects, such as (1) correlation of cell homing with positive morphological and functional effects, (2) evaluation of adverse reactions including brain hemorrhage or formation of tumors, and (3) the effect of different doses and routes of injection on cell migration and proliferation.

Studies using radiopharmaceuticals and other imaging modalities to track the fate of transplanted cells have given important clues about the mechanisms of action of cell-based therapies for neurological diseases. In many of these studies, the systemic delivery of different lineages of stem/progenitor cells exerted therapeutic effects by limiting tissue damage and/or by stimulating regeneration and plasticity of the diseased central nervous system (CNS) [58–60]. Although these effects have been associated with increased functional recovery, in most cases the number of transplanted cells that persisted at the lesion site was very low, as discussed above. These apparently paradoxical findings have shifted the attention from the controversial differentiation capacity of non-neural adult stem/progenitor cells (i.e., their potential to replace lost cells) to the mechanisms of intercellular communication between the transplanted and the host cells. Actually, even the therapeutic efficacy of cells committed to the neural fate, such as neural stem/progenitor cells, seems to be at least partially related to the secretion of innumerous neurotrophic, anti-inflammatory, and proangiogenic factors that contribute to CNS repair [61, 62]. Other potentially protective mechanisms that remain to be further characterized in the CNS are cell fusion [63] and mitochondrial transfer [64] between transplanted cells and host neurons.

Some stem/progenitor cells, such as BM-MSC, can also produce and secrete microvesicles and exosomes carrying proteins, bioactive lipids, microRNA, and mRNA. These exosomes could act through a paracrine mechanism or could be used for long-distance cellular communication when released in the systemic circulation [65]. For instance, a recent study has demonstrated that the systemic administration of purified exosomes from BM-MSC promoted functional recovery in a rodent model of stroke [66]. On the other hand, it has been shown that the exposure of BM-MSCs to ischemic brain extracts increased the levels of microRNA 133b in their exosomes. Interestingly, microRNA 133b was responsible for the increased neurite outgrowth observed in cultured neurons treated with these exosomes, suggesting that the ischemic brain could provide some cues that induce a proregenerative phenotype in BM-MSC [67].

These studies indicate that pharmacological and genetic manipulations aimed at stimulating the homing and engraftment of stem/progenitor cells in the CNS could enhance their therapeutic effects, by increasing the concentration of secreted factors at the lesion site, as well as by exposing the transplanted cells to local inflammatory cues. It is also necessary to improve the engraftment and long-term survival of neural stem cells (and their differentiated daughter cells) in some conditions where the main outcome depends on remyelination or on the replacement of lost cells, such as in congenital demyelinating diseases and in Parkinson's disease, respectively. For this purpose it will be important to investigate the signaling mechanisms regulating the expression of chemokine receptors and adhesion molecules involved in the trafficking of these cells to inflammatory sites [68–70] and to understand how the diseased CNS environment modulate the phenotype and viability of the transplanted cells. For instance, BM-MSC can adopt different phenotypes when stimulated by proinflammatory cytokines, toll-like receptors agonists or when exposed to a hypoxic culture environment* in vitro* [71]. However, it is unclear how these factors could affect the secretome of transplanted stem/progenitor cells* in vivo*. Nuclear medicine imaging and other neuroimaging methods are therefore important tools to investigate all these questions.

Another line of evidence indicates that cell-based therapies could also exert their beneficial effects at long distances from the lesion site. Large numbers of injected cells migrate to the liver and spleen, where they could interact with inflammatory cells, such as splenocytes, invariant natural killer T cells, and tissue macrophages [72, 73]. Although still poorly understood, these interactions result in the modulation of systemic inflammation and seem to be necessary for the therapeutic effects following the administration of stem/progenitor cells in models of stroke, experimental autoimmune encephalomyelitis, and traumatic brain injury [74–85]. In the latter, intravenously transplanted bone marrow-derived progenitors promoted the preservation of splenic mass, by increasing the survival and the proliferation of splenocytes [81]. This treatment also stimulated the production of the anti-inflammatory cytokine IL-10 by splenocytes and increased the percentage of T regulatory cells in the spleen and blood, which are known for their immunosuppressive effects [80, 81].

As reviewed above, in many cases systemically transplanted stem/progenitor cells can be trapped in the lungs. The pulmonary passage may represent a barrier for the delivery of these cells into the CNS [86] but could also be necessary for the activation of some cell types, before their migration to the brain, as occurs with potentially autoaggressive T cells in experimental autoimmune encephalomyelitis [87]. Indeed, Lee et al. [88] observed the upregulation of the anti-inflammatory protein tumor necrosis factor-alpha stimulated gene/protein 6 (TGS-6) in intravenously infused MSC trapped in the lungs, in a mouse model of acute myocardial infarct. Their study indicated that MSC were activated in the lungs to secrete TGS-6, which in turn modulated the inflammatory response in the heart. Moreover, it has been shown that BM-MSC induced the production of the anti-inflammatory cytokine IL-10 by macrophages from septic lungs [89]. Finally, it remains to be investigated whether stem cells could exert a beneficial effect in the lungs in some neurological conditions like stroke, which is commonly associated with pneumonia [90, 91].

Another point that remains to be investigated is the combined use of endogenous labeling of other stem cell targets. For example, activated microglia play a central part in neuroinflammation and express a protein named the translocator protein 18 kDa (TSPO) [92–95]. Augmented TSPO expression has been demonstrated in different diseases such as Alzheimer's disease, multiple sclerosis, encephalitis, and stroke [92–96]. PK11195 is a ligand of TSPO, and carbon-11 labeled PK11195 (^11^C-PK11195) is a PET radiotracer that has been used for more than 20 years to quantify TSPO expression in the brain [92, 94]. Nevertheless, it has limitations such as high nonspecific binding and new tracers such as ^11^C-PBR28 [97], 11C-DPA713 [98], and ^18^F-FEPPA [99] are under investigation.

In an example of combined use of labeling, Cicchetti et al. [100] labeled subventricular zone- (SVZ-) derived neural stem/progenitor cells with SPIOs to evaluate their migration with MRI and simultaneously utilized different radiotracers to analyze physiological aspects with PET. In their study, SVZ cells were transplanted into either the right rostral migratory stream (RMS) or striatum of adult Sprague-Dawley rats. While little migration of the cells transplanted into the striatum was seen after 3 weeks, neural stem/progenitor cells transplanted into the RMS migrated toward the olfactory bulb after 1 week. Interestingly, PET with ^18^F-FDG showed increased glucose metabolism and higher uptake of ^11^C-CFT (dopamine transporter) and ^11^C-raclopride (dopamine receptor type 2) in the striatum 3 months after the transplant. Moreover, transplanted cells did not lead to a significant local inflammatory response as evaluated by 11C-PK11195 PET. Therefore, the combination of different imaging modalities allowed cell tracking in conjunction with the assessment of cell metabolism noninvasively. Taken together, such methods have the potential of answering important questions in the field of stem cell therapy.

## 7. Conclusion

Radiopharmaceutical cell labeling is a well-established technique that allowed tracking of stem cells in preclinical and clinical studies for neurological diseases. Intralesional transplantation of radiopharmaceutically labeled stem cells led to accumulation in the site of injection in animal models of spinal cord injury. Similar findings occurred after intracerebral injection in animal models of traumatic brain injury and MCAO. In contrast, intravenous and intra-arterial injection led to small homing to the brain or spinal cord, and biodistribution was mainly to the liver, spleen, lungs, and kidneys, what also occurred in the clinical trials for stroke. Further studies are necessary to improve the understanding of the mechanisms behind cell homing and their correlation with the morphological and functional effects of stem cell therapies.

## Figures and Tables

**Table 1:** Preclinical studies using radiopharmaceuticals for stem cell tracking in neurology.

Study reference	Radiopharmaceutical	Model	Animals	Cell type	Route	Time from lesion	Number of treated animals (number of controls)	Number of cells injected	Infusion volume, rate, and duration	Imaging time points
de Haro et al., 2005 [19]	^111^In-oxine	Spinal cord injury at T8-T10	Wistar rats	Rat BM-MSCs	Tail vein or intralesional	3 months	20 (no controls)	6 × 10^6^	0.1 or 1 mL	3–10 days
Mäkinen et al., 2006 [20]	^111^In-oxine	Transient MCAO (120 minutes)	Wistar rats	Human UCB-MNCs	Femoral vein	24 h	Not specified	1–7 × 10^6^	0.5 mL	0 and 24 h
Lappalainen et al., 2008 [21]	^111^In-oxine	Transient MCAO (120 minutes)	Wistar rats	Human ES-NPCs or rat HPCs	Common carotid artery or femoral vein	24 h	13 (controls not specified)	1 × 10^6^	0.5 mL	0 and 24 h
Lo et al., 2008 [22]	^131^I-FIAU	Spinal cord injury at T10	Long-Evans rats	Mouse embryo-derived fibroblasts	Spinal cord at L1 level	0 h	20 (no controls)	1 × 10^6^	0.5 mL	2, 24 and 48 h
Detante et al., 2009 [23]	^99m^Tc-HMPAO	Transient MCAO (90 minutes)	Sprague-Dawley rats	Human BM-MSCs	Saphenous vein	7 days	9 (9 controls)	3.4 ± 1.2 × 10^6^	1 mL	2 and 20 h
Yoon et al., 2010 [24]	^111^In-oxine	Traumatic brain injury	Sprague-Dawley rats	Rat BM-MSCs	Tail vein	24 h	3 (2 controls)	1 × 10^6^	1 mL	24 h
Park et al., 2011 [25]	^99m^Tc-HMPAO	Traumatic brain injury	Sprague-Dawley rats	Rat BM-MSCs	Tail vein	Not specified	14 (13 controls)	1 × 10^6^	1 mL	4 h
Vasconcelos-dos-Santos et al., 2012 [26]	^99m^Tc	Permanent thermocoagulation of pial blood vessels	Wistar rats	Human BM-MNCs	Common carotid artery or jugular vein	24 h	12 (8 controls)	3 × 10^7^	0.5 mL	2 and 24 h
Arbab et al., 2012 [27]	^111^In-oxine	Transient MCAO (120 minutes)	Wistar rats	Human UTCs	Tail vein	48 h	13 (12 controls)	3 × 10^8^	2 mL	0, 1, and 3 days
Mitkari et al., 2013 [28]	^111^In-oxine	Transient MCAO (90 minutes)	Wistar rats	Human BM-MSCs	External carotid artery	24 h	19 (controls not specified)	0.5–1.1 × 10^6^	0.5 mL	30 minutes and 24 h
Gubert et al., 2013 [29]	^99m^Tc	Permanent bilateral common carotid ligation	Lister hooded rats	Rat BM-MNCs	Tail vein	24 h	23 (no controls)	2 × 10^7^	0.4 mL	1 h
Goldmacher et al., 2013 [30]	^111^In-oxine	Transient MCAO (120 minutes)	Sprague-Dawley rats	Rat BM-MSCs	Tail vein	24 h	3 (3 controls)	5 × 10^5^	0.5 mL	4, 20, 44, and 70 h
Makela et al., 2013 [31]	^99m^Tc-HMPAO	Transient brachiocephalic trunk and subclavian artery occlusion (7 minutes)	Pigs	Pig BM-MNCs	Brachiocephalic trunk	24 h	10 (no controls)	6–20 × 10^6^	Not specified	2, 4, 6, 12, and 24 h
Manley et al., 2013 [32]	^99m^Tc-HMPAO	Transient MCAO (60 minutes)	Sprague-Dawley rats	Rat BM-DCs	Carotid artery	3 h	4 (no controls)	2 × 10^6^	0.3 mL/0.1 mL/min	5–20 minutes and 5-6 h
Ramos et al., 2013 [33]	^99m^Tc	Permanent vertebral occlusion and transient carotid artery occlusion (17 minutes)	Wistar rats	Rat BM-MNCs	Common carotid artery	24 or 72 h	4 (5 controls)	3 × 10^7^	0.3 mL	2 h
Wu et al., 2013 [34]	^131^I-FIAU	Transient MCAO (60 minutes)	Sprague-Dawley rats	Rat BM-MSCs	Intracerebral, intraventricular, carotid artery or tail vein	24 h	20 (4 controls)	2 × 10^6^	15 *μ*L (intracerebral and intraventricular) or 0.5 mL (carotid artery or tail vein)	2, 8, and 24 h
Guan et al., 2013 [35]	^18^F-FDG	Traumatic brain injury	Sprague-Dawley rats	Human BM-MSCs and/or collagen scaffold	Intracerebral	7 days	9 (MSCs); 9 (MSCs + collagen)	3 × 10^6^	0.2 mL	3, 6, and 12 h

^111^In-oxine: indium-111-oxine; ^131^I-FIAU: iodine-131-2′-fluoro-2′-deoxy-1-*β* D-arabinofuranosyl-5-iodouracil; ^18^F-FDG: fluorine-18-fluorodeoxyglucose; ^99m^Tc: technetium-99m; ^99m^Tc-HMPAO: technetium-99m-hexamethylpropyleneamine oxime; BM-DCs: bone marrow-derived dendritic cells; BM-MNCs: bone marrow mononuclear cells; BM-MSCs: bone marrow-derived mesenchymal stem cells; EPCs: endothelial progenitor cells; ES-NPCs: embryonic stem cell-derived neural progenitor cells; HPCs: hippocampal progenitor cells; MCAO: middle cerebral artery occlusion; UCB-MNCs: umbilical cord blood mononuclear cells; UTCs: umbilical tissue-derived cells.

**Table 2:** Clinical studies using radiopharmaceuticals for stem cell tracking in neurology.

Study reference	Radiopharmaceutical	Route	Cell type	Type of lesion	Time from lesion	Number of treated patients (number of controls)	Number of cells injected	Infusion volume, rate, and duration	Imaging time points
Correa et al., 2007 [36]	^99m^Tc-HMPAO	Middle cerebral artery	BM-MNCs	Middle cerebral artery ischemic stroke	9 days	1 (no controls)	3 × 10^7^	Not specified	8 h
Barbosa da Fonseca et al., 2009 [37], 2010 [38]; Battistella et al., 2011 [39]; Rosado-de-Castro et al., 2013 [40]	^99m^Tc	Middle cerebral artery or cephalic vein	BM-MNCs	Middle cerebral artery ischemic stroke	19–89 days	12 (no controls)	1 × 10^8^ to 5 × 10^8^	10 mL in 10 min (1 mL/min)	2 and 24 h

^99m^Tc: Technetium-99m; ^99m^Tc-HMPAO: Technetium-99m-hexamethylpropyleneamine oxime; BM-MNCs: bone marrow mononuclear cells.
